# LncRNA FENDRR Inhibits ox-LDL Induced Mitochondrial Energy Metabolism Disorder in Aortic Endothelial Cells *via* miR-18a-5p/PGC-1*α* Signaling Pathway

**DOI:** 10.3389/fendo.2021.622665

**Published:** 2021-04-12

**Authors:** Guiming Wang, Yudong Yang, Honggang Ma, Liuming Shi, Wenbin Jia, Xing Hao, Weizong Liu

**Affiliations:** Department of Vascular Surgery, The First Hospital of Shanxi Medical University, Taiyuan, China

**Keywords:** atherosclerosis, mitochondrial dysfunction, endothelial cell, FENDRR, miR-18a-5p, PGC-1*α*

## Abstract

Atherosclerosis (AS) is the main cause of morbidity and mortality in the world. Mitochondrial dysfunction is closely related to AS. At present, several signaling pathways related to mitochondrial dysfunction have been found, one of which is around PGC-1*α*. PGC-1*α* is a transcription activator, which is related to mitochondrial biogenesis and antioxidant defense. In this study, we explored the effect of miR-18a-5p/PGC-1*α* signaling pathway on mitochondrial energy metabolism in HAECs with ox-LDL treatment. The results showed that the mitochondrial energy metabolism disorder in HAECs treated by ox-LDL was related to the downregulation of LncRNA FENDRR and PGC-1*α*. FENDRR could reverse ox-LDL induced mitochondrial energy metabolism disorder and upregulate the PGC-1*α* expression. FENDRR could be used as ceRNA to inhibit the miR-18a-5p expression and reduce the negative regulation of miR-18a-5p on PGC-1*α*. Downregulation of miR-18a-5p expression or upregulation of PGC-1*α* in ox-LDL treated HAECs could reverse mitochondrial energy metabolism disorder. In conclusion, these findings suggested that FENDRR/miR-18a-5p/PGC-1*α* signaling pathway regulated mitochondrial energy metabolism in HAECs; ox-LDL downregulated the expression of PGC-1*α* and cause mitochondrial energy metabolism disorder by inhibiting this signal pathway.

## Introduction

Atherosclerosis (AS), which leads to ischemia–reperfusion (I/R) injury of the heart, brain, and other tissues, is a chronic inflammatory disease and is an important risk factor for premature death (1). AS plays a key role in the pathogenesis of cardiovascular diseases. The course of AS is complex and is mainly manifested by the highly expressed adhesion molecules of arterial endothelial cells; they promote the infiltration of monocytes and differentiate into highly active lipid foam cells, accompanied by the migration of vascular smooth muscle cells (VSMCs) into the intima (1, 2). AS is caused by the long-term and excessive reaction of vascular wall to injury; it starts from oxidative damage and inflammatory changes of endothelial cells (3). The apoptosis of vascular endothelial cells caused by mitochondrial ROS disorder is the initial stage of AS (4). In vascular disorder process, mitochondrial ROS increased in endothelial cells induced by a variety of factors, such as hypertension, hyperglycemia, and hyperlipidemia (5). Oxidized low-density lipoprotein (ox-LDL) regulates the migration, apoptosis, proliferation, and differentiation of vascular endothelial cells (VECs) (6). ox-LDL induced oxidative stress of VEC by promoting the production of ROS (7). There are many sources of ROS production in cells, such as mitochondria, NADPH oxidase or xanthine oxidase (XO), but the mitochondria have been considered as the major source of ROS in human endothelial cells. ROS produced by the mitochondria is the main promoter of cell signal under stress stimulation or risk factor exposure (8).

Peroxisome Proliferator Activated Receptor (PPAR) *γ*-Coactivator 1-*α* (PGC-1*α*), a transcription factor, is involved in the upstream regulation of lipid catabolism, mitochondrial function. It is also a key molecule that regulates mitochondrial energy metabolism (9). Valle reported that an increased PGC-1*α* expression in human umbilical vein endothelial cells reduces ROS level, increases mitochondrial membrane potential, and reduces apoptosis under oxidative stress (10). PGC-1*α* could also resist excessive oxidative stress by increasing the transcription of antioxidant genes, containing manganese superoxide dismutase (MnSOD) (11). PGC-1*α* could restore the physiological membrane potential by increasing the activity of ATP/ADP transposase and inhibiting the production of excessive ROS (12). These results suggested that PGC-1*α* played an important role in regulating mitochondrial energy metabolism of VECs.

Non-coding RNAs include miRNA, piRNA, tRNA, and lncRNA (13–15). They do not have a coding function. However, they have a transcriptional regulation function and play a variety of roles. miRNA is a small RNA with a length of about 22 nt. It can bind with 3′UTR of mRNA through base complementary pairing, inducing mRNA splicing or translation block and resulting in gene silencing so as to regulate gene expression (16). Some lncRNAs regulate gene expression by recruitment of transcriptional regulatory complexes to the proximal (CIS) or distal (trans) genomic binding sites; others can be used as scaffold or decoys to assist protein or miRNA binding (17). Many studies have shown that a variety of miRNAs and lncRNAs were involved in the regulation of VEC function (18–20).

In recent years, some lncRNAs have been found to be differentially expressed in human cardiovascular diseases, which can be used as predictors of cardiovascular disease (21). FENDRR is an important lncRNA related to cardiac development and pathology; it is the regulator of two important histone modified complexes PRC2 and TrxG/MLL, which induces histone H3 lysine 27 trimethylation (H3K27Me3) by binding to PRC2 complex and inhibits many gene expressions at the transcriptional level (22). The expression level of FENDRR decreased in atherosclerotic plaques, which may promote the VSMC proliferation through the decrease of H3K27Me3 (23). FENDRR is a tumor suppressor gene in the tumor, which is related to the proliferation, invasion, and metastasis of tumor cells. In gastric cancer, the FENDRR expression decreased and caused the increased expression of fibronectin, which increased the invasion and metastatic ability of gastric cancer cells (24). FENDRR inhibited the progression of NSCLC by regulating TIMP2 expression through binding with miR-761 (25). FENDRR may play a role of tumor suppressor gene by inhibiting SOX4 in colon cancer (26). In addition, the expression level of FENDRR was decreased in primary liver cancer, chronic myeloid leukemia, osteosarcoma, prostate cancer, renal cell carcinoma, and cholangiocarcinoma, leading to the upregulation of tumor cell proliferation, invasion, and metastasis (27–32). To our knowledge, the role of FENDRR in aortic endothelial cells has not been reported.

In this study, we speculated that ox-LDL may cause dysfunction of endothelial cells by affecting the expression of PGC-1*α*. We analyzed miRNA and lncRNA that may regulate PGC-1*α* expression by ENCORI (33) and explored the effect of FENDRR/miR-18a-5p/PGC-1*α* regulatory axis on mitochondrial energy metabolism in HAECs with ox-LDL treatment.

## Materials and Methods

### Cells

HAECs were purchased from ATCC (Manassas, VA, USA). They were cultured with DMEM/F12 medium containing FBS (10%), 1% penicillin and streptomycin at 37°C, 5% CO2. ox-LDL (final concentration 50 μg/ml) was added into the ox-LDL treated group. The cells were subcultured by not more than five times in our laboratory. This study was approved by the biosafety committee of the First Hospital of Shanxi Medical University and complied with biosafety procedures rules.

### Cell Transfection

lncRNA FENDRR shRNA (1.25 μg/ml), PGC-1*α* shRNA (1.25 μg/ml), miR-18a-5p mimics and miR-18a-5p inhibitor (37.5 pmol/ml) were obtained from GenePharma. A pair of complementary oligonucleotide sequences was designed for FENDRR and PGC-1*α* respectively. sh-FENDRR-F: AAGCTGCGATTGACTGTCTTATAACTCGAG TTATAAGACAGTCAATCGCTTTTT G; sh-FENDRR-R: GATCC AAAAAGCGATTGACTGTCTTATAA CTCGAG TTATAAGACAGTCAATCGCA; sh-PGC-1*α*-F:AAGCTGCGAATCCAGTTTGTGCAACTCGAG TGCACAAACTGGATTCGCTTTTT G; sh-PGC-1*α*-R:GATCC AAAAAGCGAATCCAGTTTGTGCAA CTCGAG TTGCACAAACTGGATTCGC A. They were cloned to pcDNA3.1 vector. Lipofectamine 3000 kit (Invitrogen, Carlsbad, CA, USA) was used for transfection according to the manual. Briefly, 2.5 μg shRNA or eukaryotic expression plasmid was mixed with 125 μl Opti-MEM medium; 7.5 μl Lipofectamine 3000 was mixed with 125 μl Opti-MEM medium and incubated at room temperature for 5 min. They were mixed and incubated at room temperature for 5 min to form plasmid/Lipofectamine 3000 complex and then added to the cells cultured in six-well plate for 24 h. Similarly, 75 pmol miR-18a-5p mimics or miR-18a-5p inhibitor was mixed with 125 μl Opti-MEM medium; 7.5 μl Lipofectamine 3000 was mixed with 125 μl Opti-MEM medium and incubated at room temperature for 5 min. They were mixed and incubated at room temperature for 5 min to form plasmid/Lipofectamine 3000 complex and then added to the cells cultured in six-well plate for 24 h.

### Cell Apoptosis Detection

Apoptosis detection was performed using AnnexinV-PI Analysis Kit (Beyotime Biotechnology, Shanghai, China) following the manufacturer’s instructions. The cells were inoculated in a six-well plate. ox-LDL (12.5 μl and final concentration was 50 μg/ml) was added to the ox-LDL treatment group when the cells grew to 70% confluent. Then the same amount of solvent was added to the control group and cultured for 48 h. Cells were collected, digested, and washed three times with ice-cold PBS. 1 × 10^6^ cells/ml were collected and resuspended in 300 μl 1× binding buffer. 195 μl Annexin V-FITC, 5 μl Annexin V-FITC, and 10 μl PI were added according to the kit’s protocol. Pre-cooled 1× binding buffer (200 μl) was added after 10 min of incubation at 4°C refrigerator in the dark. The apoptosis rate was detected using flow cytometry. The results were analyzed by CELLQUEST software (BD Biosciences). All tests were repeated three times.

### Mitochondrial ROS Determination

The cells were inoculated in a six-well plate. ox-LDL (12.5 μl and final concentration was 50 μg/ml) was added to the ox-LDL treatment group when the cells grew to 70% confluent. Then the same amount of solvent was added to the control group and culture for 48 h. The cells were cultured at 37°C with fresh media containing 5 μM MitoSox Red mitochondrial superoxide indicator (Invitrogen) for 10 min. The cells were washed in PBS, and then mean fluorescence intensity (MFI) was detected by flow cytometry (FACS Aria, Becton Dickinson, San Jose, CA). All tests were repeated three times.

### Mitochondrial Complexes Activity

The cells were inoculated in a six-well plate. ox-LDL (12.5 μl and final concentration was 50 μg/ml) was added to the ox-LDL treatment group when the cells grew to 70% confluent. Then the same amount of solvent was added to the control group and culture for 48 h. Mitochondria were first isolated from cultured cells using Mitochondria Isolation Kit for cultured cells following the manufacturer’s instructions. Activities of respiratory chain complexes in the mitochondria were measured in microplates using the specific kits complex I–IV from Abcam (Cambridge, UK) normalized to protein concentration as measured by DC Protein Assay (BioRad). All tests were repeated three times.

### Mitochondrial DNA Estimation

The cells were inoculated in a six-well plate. ox-LDL (12.5 μl and final concentration was 50 μg/ml) was added to the ox-LDL treatment group when the cells grew to 70% confluent. Then the same amount of solvent was added to the control group and culture for 48 h. Mitochondrial DNA copy number was estimated as previously described (34). Briefly, the total DNA was isolated with DNeasy Tissue Kit (Qiagen) according to instructions. Quantitative PCR was performed by using an equal amount of DNA from each sample, Power SYBR Green Master Mix (LifeTec Garland Island, NY), and 5 μM of each primer, *i.e.* mitochondrial ND1, mitochondrial Cyt B, and nuclear H19 DNA. To compare mitochondrial copy number, ratio of ND1:H19 and/or Cyt B:H19 was calculated. Results are presented as percent change in the mitochondrial DNA in the experimental *versus* wild-type/control samples. All tests were repeated three times.

### Mitochondrial ATP Determination

The cells were inoculated in a six-well plate. ox-LDL (12.5 μl and final concentration was 50 μg/ml) was added to the ox-LDL treatment group when the cells grew to 70% confluent. Then the same amount of solvent was added to the control group and culture for 48 h. ATP levels were determined using the Cell Titre-Glo Luminescent Cell Viability Assay Kit (Promega), as previously described (35). All tests were repeated three times.

### Seahorse Metabolic Analyzer Assays

Cell energy metabolism analysis was performed on cell energy metabolism analyzer (Seahorse, USA). The cells in different groups were inoculated (5 × 10^4^ cells/well) to 24 well XF24 plate (Seahorse Biosciences). Cartridge plates for metabolic stress injections were hydrated with calibrant solution (Seahorse Biosciences) for at least 8 h at 37°C prior to the assay. One hour before detection, the medium of each well was aspirated, and the cells were washed twice with 1 ml of XF detection solution (Seahorse Biosciences). The detection solution was added to each well until a final volume of 500 μl/well was achieved. The cell plate was placed in an incubator (CO_2_ free) at 37°C for 1 h. Calibrant solution hydrated for 8 h was added to each well. Assay conditions and set-up were performed according to instructions. Oxygen consumption rate (OCR) was reported in the unit of picomoles per minute. The results were normalized to cell number using CyQUANT Direct Cell Proliferation Assay kit (Molecular Probes). The area under curve (AUC) was calculated as previously described (36). All tests were repeated three times.

### Interaction of miRNA and lncRNA

miRWalk 3 is a comprehensive database of miRNA target genes. ENCORI (The Encyclopedia of RNA Interactomes) is mainly focus on miRNA–target interactions (33). We used ENCORI (starbase.sysu.edu.cn) and miRWalk 3 (http://mirwalk.umm.uni-heidelberg.de/) to analyze all miRNAs that could bind to FENDRR and PGC-1*α* 3′-UTR.

### RT-PCR

Total RNA was extracted by TRIzol reagent according to the instructions. The purity and content of RNA were determined by NanoDrop™ 1000 spectrophotometer. MMLV Reverse Transcriptase kit (Takara Bio Inc) was used for reverse transcription according to the manual. miR-18a-5p mRNA expression was detected by GenePharma Hairpin-it TM microRNA RT-PCR Quantitation Kit according to the instructions. The thermocycler conditions were 95°C for 10 min, 40 cycles of 95°C for 12 s and 62°C for 40 s. lncRNA FENDRR and PGC-1*α* mRNA expression was detected by SYBR Premix Ex Taq™ Kit (Takara Bio Inc). GAPDH and U6 were used as internal control. Quantifications was performed by the2^−△△^Ct method. Primers’ sequences were listed in **Table 1**.

**Table 1:** Primers used in this study.

Gene	Primer name	Primer sequence(5′–3′)
hsa-miR-15a-5p	RT primer	GTCGTATCCAGTGCAGGGTCCGAGGTATTCGCACTGGATACGACCACAAA
	Forward primer	CGCGTAGCAGCACATAATGG
	Reverse primer	AGTGCAGGGTCCGAGGTATT
hsa-miR-16-5p	RT primer	GTCGTATCCAGTGCAGGGTCCGAGGTATTCGCACTGGATACGACCGCCAA
	Forward primer	CGCGTAGCAGCACGTAAATA
	Reverse primer	AGTGCAGGGTCCGAGGTATT
hsa-miR-214-3p	RT primer	GTCGTATCCAGTGCAGGGTCCGAGGTATTCGCACTGGATACGACACTGCC
	Forward primer	GCGACAGCAGGCACAGACA
	Reverse primer	AGTGCAGGGTCCGAGGTATT
hsa-miR-15b-5p	RT primer	GTCGTATCCAGTGCAGGGTCCGAGGTATTCGCACTGGATACGACTGTAAA
	Forward primer	CGCGTAGCAGCACATCATGG
	Reverse primer	AGTGCAGGGTCCGAGGTATT
hsa-miR-195-5p	RT primer	GTCGTATCCAGTGCAGGGTCCGAGGTATTCGCACTGGATACGACGCCAAT
	Forward primer	GCGCGTAGCAGCACAGAAAT
	Reverse primer	AGTGCAGGGTCCGAGGTATT
hsa-miR-18a-5p	RT primer	GTCGTATCCAGTGCAGGGTCCGAGGTATTCGCACTGGATACGACCAACAA
	Forward primer	CGCGTCCAGCATCAGTGATT
	Reverse primer	AGTGCAGGGTCCGAGGTATT
U6	RT primer	GTCGTATCCAGTGCAGGGTCCGAGGTATTCGCACTGGATACGACAAAATATGGAAC
	Forward primer	CTCGCTTCGGCAGCACA
	Reverse primer	AACGCTTCACGAATTTGCGT
FENDRR	Forward primer	CACCACAGGTCCAAGTAG
	Reverse primer	TTTAACGATCCCACCAAC
PPARGC1A	Forward primer	TCAAGCCACTACAGACACC
	Reverse primer	CTGCGATATTCTTCCCTC
GAPDH	Forward primer	TGGGTGTGAACCACGAGAA
	Reverse primer	GGCATGGACTGTGGTCATGA

### Western Blot

The cells were lysed by Cell Lysis Solution. They were centrifuged at 4°C (1,000 rpm) for 5 min; the supernatant was collected. Proteins were extracted. Their concentration was measured with BCA. Proteins (50 μg/lane) were separated with 10% SDS−PAGE, then they were transferred to a membrane. The PVDF membrane was washed and blocked by buffer. PGC-1*α* antibody (Novus Biologicals, LLC, Centennial, CO, USA, NBP1-04676, 1:1,000) and *β*-actin antibody (Abcam, Cambridge, UK, ab8226, 1:2000) were added, and they were incubated at 4°C overnight. The membrane was rinsed, and the secondary antibody was added; they were incubated at RT for 1 h. They were determined by an enhanced chemiluminescence kit (Perkin-Elmer Inc., Waltham, MA, USA). They were quantified with Imagequant LAS4000 (GE Healthcare, Japan).

### Double Luciferase Reporter Gene Assay

Wild type wt-pGL3-FENDRR and mutant type mut-pGL3-FENDRR plasmids were constructed based on the prediction. FENDRR luciferase reporter gene, miR-18-5p, and Renilla luciferase were co-transfected HEK293 cells. They were split by Dual Luciferase Assay following the protocol after they were cultured for 48 h. The results were analyzed by Panomics Luminometer. They were homogenized by sea renin fluorescence.

For analyzing the action between miR-18-5p and PGC-1*α* 3′-UTR, wild and mutant type PGC-1*α* 3′-UTR luciferase reporter gene plasmid wt-pGL3-PGC-1*α* and mut-pGL3-PGC-1*α* were constructed respectively. The luciferase reporter gene plasmids, miR-18-5p, and Renilla luciferase were co-transfected HEK293 cells. The analysis is the same as the above method.

### RNA Binding Protein Immunoprecipitation Assay

RIP experiments were conducted by EZMagna RIP kit (Catalog No. 17-701, Millipore, Billerica, MA, USA) according to the instruction. The cells were lysed when they grew to 80–90% full. Then cell lysate (100 μl) was incubated with anti-Ago2 antibody bound magnetic beads, normal mouse IgG (negative control) or anti-SNRNP70; they were incubated at 55°C for 30 min to remove protein using proteinase K. RNA was purified with RNeasy Micro Kit (Qiagen, Dusseldorf, Germany). The content and purity of RNA were detected by NanoDrop 1000 spectrophotometer. They were detected by RT-PCR.

### Statistical Analyses

The statistical tests were conducted by SPSS 20.0 software. The differences among groups were tested by one-way ANOVA or Student’s t-test. The correlation was examined by Spearman correlation test. *P* < 0.05 was considered statistically significant.

## Results

### ox-LDL Caused Mitochondrial Energy Metabolism Disorder, Upregulate Mitochondrial ROS Level and Apoptosis, and Inhibit the Expression of LncRNA FENDRR and PGC-1*α*

**Figure 1:**
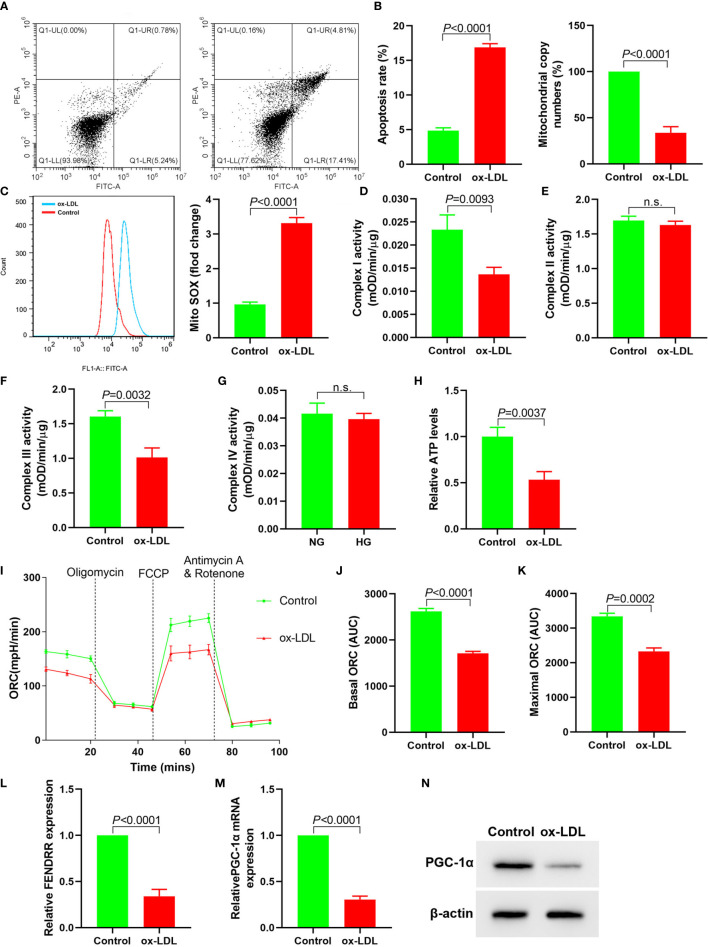
ox-LDL inhibited mitochondrial energy metabolism and promoted mitochondrial ROS accumulation and apoptosis in HAECs (N = 3). **(A)** Apoptosis of HAECs detected by flow cytometry; **(B)** RT-PCR results of the copy number of mtDNA in HAECs; **(C)** Mitochondrial ROS levels in HAECs detected by flow cytometry; **(D–G)** Mitochondrial complex I, II, III and IV activity in HAECs; **(H)** Relative ATP content in HAECs; **(I–K)** Mitochondrial OCR analysis of ox-LDL treated HAECs using the Seahorse XF24 Bioanalyzer device. A total of 56 μl 10×Oligomycin solution (final concentration was 2 μM), 62 μl 10× FCCP solution (final concentration was 2 μM), and 69 μl 10× rotenone (final concentration was 0.5 μM)/antimycin A solution (final concentration was 0.5 μM) were added at the times indicated by dashed lines. Basal and maximal respiratory rates of different groups were determined by calculating the area under curve (AUC); **(L)** RT-PCR results FENDRR mRNA expression in HAECs; **(M)** RT-PCR results of ox-LDL on PGC-1*α* mRNA expression in HAECs; **(N)** Western blotting results of PGC-1*α* protein expression in HAECs. ox-LDL caused mitochondrial energy metabolism disorder and up regulated mitochondrial ROS level and apoptosis in HAECs. n.s., No significance.

In order to observe the effect of ox-LDL on mitochondrial function of HAECs, we analyzed the changes of mitochondrial function of HAECs treated with ox-LDL. ox-LDL also reduced the copy number of mitochondrial DNA in HAECs (**Figure 1B**) and inhibited the activities of complexes I and III (**Figures 1D, E** **)**, but it had no effect on the activities of complexes II and IV (**Figures 1F, G**). ox-LDL decreased ATP levels in HAECs (**Figure 1H**). The basic and maximal oxygen consumption rates (OCRs) in HAECs treated by ox-LDL were significantly decreased (**Figures 1I–K**). The expression levels of FENDRR and PGC-1*α* in HAECs decreased significantly after ox-LDL treatment (**Figures 1L–N**). These results suggested that ox-LDL caused mitochondrial energy metabolism disorder, upregulated mitochondrial ROS level and apoptosis in HAECs (**Figures 1A, C**), which may involve the regulation of FENDRR and PGC-1*α* expression.

### LncRNA FENDRR Reversed ox-LDL Induced Mitochondrial Energy Metabolism Disorder and Upregulated PGC-1*α* Expression in HAECs

**Figure 2:**
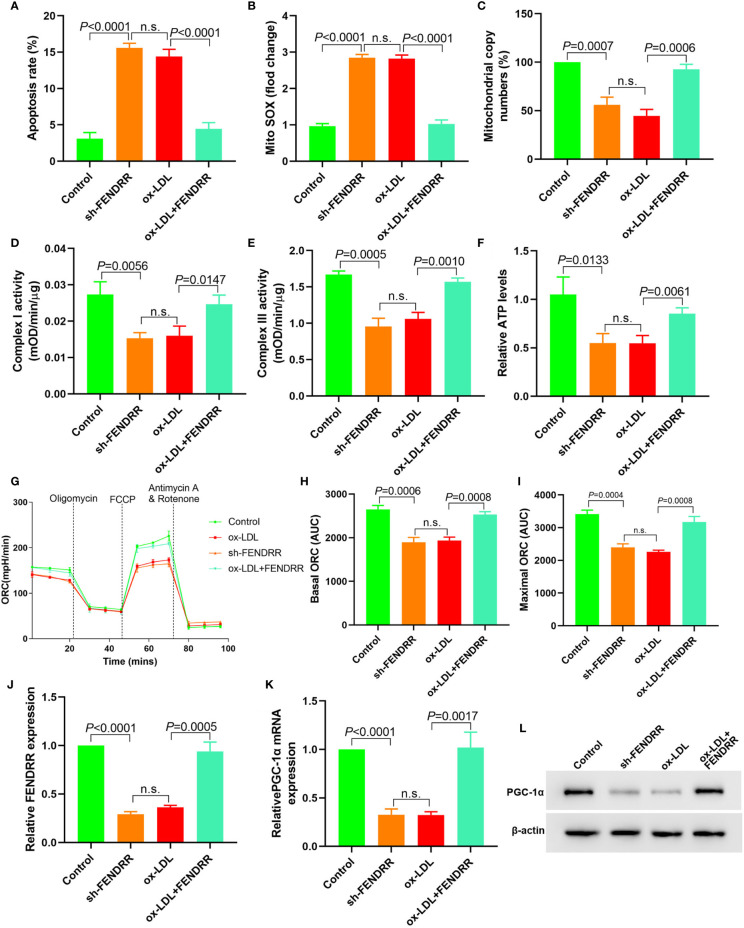
LncRNA FENDRR reversed ox-LDL induced mitochondrial energy metabolism disorder and upregulated PGC-1*α* expression in HAECs. **(A)** Cell apoptosis was detected by flow cytometry; **(B)** Mitochondrial ROS levels detected by flow cytometry; **(C)** mtDNA copy number changes detected by RT-PCR; **(D, E)** The activity of mitochondrial complexes I and III; **(F)** Relative ATP levels; **(G–I)** Mitochondrial OCR analysis of different groups using the Seahorse XF24 Bioanalyzer device; **(J)** FENDRR expression detection; **(K)** RT-PCR results of PGC-1*α* mRNA expression; **(L)** Western blotting results of PGC-1*α* protein expression. LncRNA FENDRR reversed ox-LDL induced mitochondrial energy metabolism disorder and upregulated PGC-1*α* expression in HAECs. n.s., No significance.

Downregulation of FENDRR expression in HAECs promoted apoptosis (**Figure 2A**), decreased mtDNA copy number (**Figure 2B**), and resulted in mitochondrial ROS accumulation (**Figure 2C**). Downregulation of FENDRR expression also inhibited complex I (**Figure 2D**) and III activity (**Figure 2E**) and downregulated ATP level (**Figure 2F**), which were the same as that of ox-LDL treatment. The basic and maximal OCRs in HAECs were significantly decreased after FENDRR was downregulated (**Figures 2G–I**). FENDRR overexpression reversed the regulation of ox-LDL on mitochondrial energy metabolism in ox-LDL treated HAECs. PGC-1*α* expression was downregulated in HAECs after FENDRR was downregulated and *vice versa* (**Figures 2J–L**). These results suggested that LncRNA FENDRR reversed ox-LDL induced mitochondrial energy metabolism disorder and upregulated PGC-1*α* expression in HAECs.

### LncRNA FENDRR Could Be Used as ceRNA to Adsorb miR-18a-5p and Upregulate the PGC-1*α* Expression

**Figure 3:**
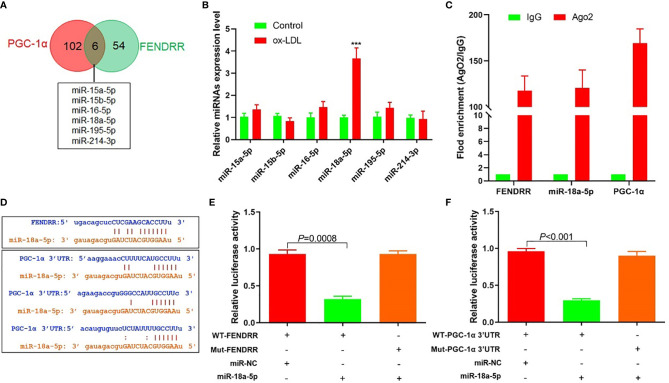
FENDRR acted as ceRNA to regulate the expression of miR-18a-5p and PGC-1*α.* **(A)** Bioinformatics predicted that six miRNAs could simultaneously bind with FENDRR and PGC-1*α* 3′-UTR; **(B)** RT-PCR results of six miRNAs’ expression; **(C)** RIP assay was used for detection of the enrichment of miR-18a-5p, FENDRR, and PGC-1*α* in response to anti-Ago2 compared to the negative control IgG; **(D)** The binding site of miR-18a-5p, FENDRR, and PGC-1*α* 3′-UTR; **(E)** Targeting effect of miR-18a-5p and FENDRR; **(F)** Targeting effect of miR-18a-5p and PGC-1*α* 3′-UTR. FENDRR acted as a ceRNA to inhibit miR-18a-5p level, further promoted the PGC-1*α* expression, and maintained the stability of mitochondrial energy metabolism in HAECs.

The above results suggested that there may be a direct regulatory relationship between FENDRR and PGC-1*α*. Six miRNAs were found to bind to FENDRR and PGC-1*α* 3′-UTR simultaneously (**Figure 3A**). RT-PCR was used to detect the expression of these six miRNAs in HAECs after ox-LDL treatment. The results showed that the miR-18a-5p expression level increased most significantly after ox-LDL treatment (**Figure 3B**). RIP assay showed that miR-18a-5p, FENDRR, and PGC-1*α* mRNA were enriched in the precipitate (**Figure 3C**), which indicated that there was a direct combination of the three. Further analysis of gene sequences showed that there were target regulatory sites between FENDRR and miR-18a-5p and between miR-18a-5p and PGC-1*α* 3′-UTR (**Figure 3D**). The results of luciferase assay also showed that FENDRR and miR-18a-5p (**Figure 3E**) and miR-18a-5p and PGC-1*α* 3′-UTR (**Figure 3F**) had targeted regulatory effects. These results suggested that FENDRR, as a ceRNA, inhibited miR-18a-5p level, further promoted the PGC-1*α* expression, and maintained the stability of mitochondrial energy metabolism in HAECs.

### Downregulation of miR-18a-5p Expression in ox-LDL Treated HAECs Reversed Mitochondrial Energy Metabolism Disorder and Upregulated PGC-1α Expression

**Figure 4:**
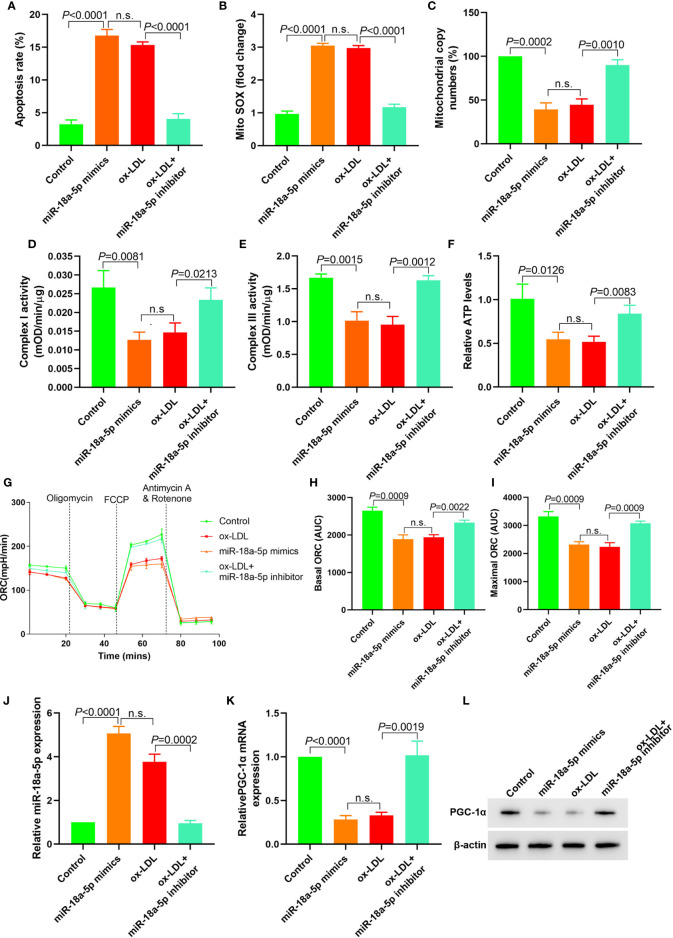
Downregulation of miR-18a-5p reversed the mitochondrial energy metabolism disorder induced by ox-LDL and upregulated the PGC-1*α* expression. **(A)** Cell apoptosis detected by flow cytometry; **(B)** Mitochondrial ROS levels detected by flow cytometry; **(C)** Changes of mtDNA copy number; **(D, E)** The activity of mitochondrial complexes I and III; **(F)** Relative ATP levels; **(G–I)** Mitochondrial OCR analysis of different groups by the Seahorse XF24 Bioanalyzer device. **(J)** miR-18a-5p expression detection; **(K)** RT-PCR results of PGC-1*α* mRNA expression; **(L)** Western blotting results of PGC-1*α* protein expression. Downregulation of miR-18a-5p expression in ox-LDL treated HAECs reversed mitochondrial energy metabolism disorder and upregulated PGC-1*α* expression. n.s., No significance.

In miR-18a-5p mimics transfected HAECs, apoptosis, and mitochondrial ROS levels were significantly increased; mitochondrial DNA copy number, complex I and III activities, and ATP levels were significantly decreased, and basic and maximal OCRs were also significantly decreased. After miR-18a-5p inhibitor transfection in ox-LDL treated HAECs, apoptosis and mitochondrial ROS levels were significantly decreased, mitochondrial DNA copy number, complex I and III activities, and ATP levels were significantly increased, and basic and maximal OCRs were also significantly increased (**Figures 4A–I**). PGC-1*α* expression was downregulated in HAECs after transfection of miR-18a-5p mimics in HAECs and *vice versa* (**Figures 4J–L**). These results suggested that downregulation of miR-18a-5p expression in ox-LDL treated HAECs reversed mitochondrial energy metabolism disorder and upregulated PGC-1*α* expression.

### Upregulation of PGC-1*α* Expression in ox-LDL Treated HAECs Reversed Mitochondrial Energy Metabolism Disorder

**Figure 5:**
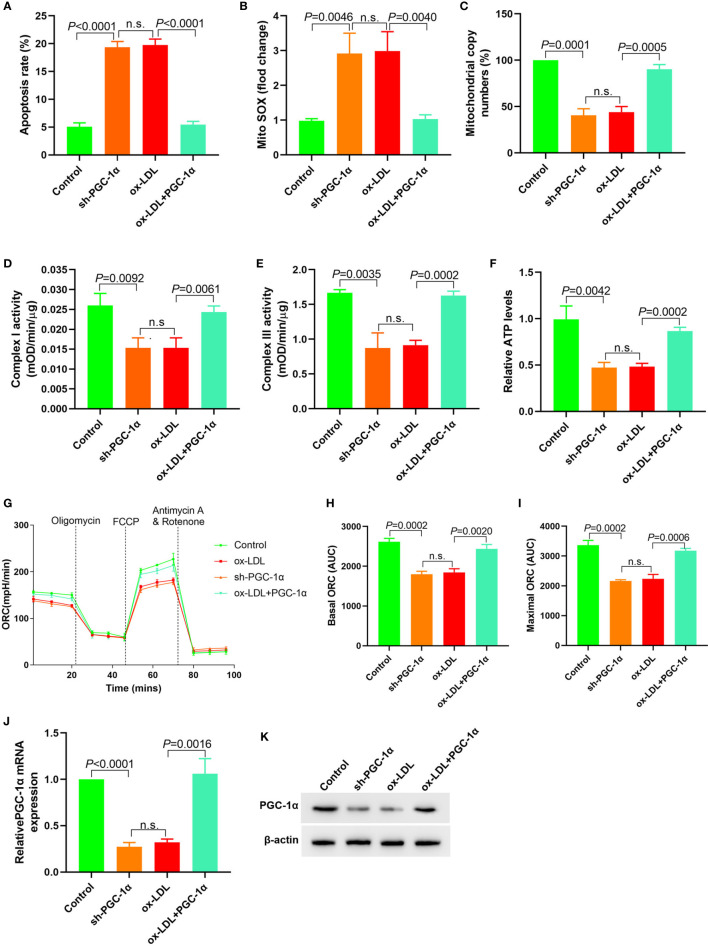
Upregulation of PGC-1*α* expression in ox-LDL treated HAECs reversed mitochondrial energy metabolism disorder **(A)** Cell apoptosis detected by flow cytometry; **(B)** Mitochondrial ROS levels detected by flow cytometry; **(C)** Changes of mtDNA copy number; **(D, E)** The activity of mitochondrial complexes I and III; **(F)** Relative ATP levels; **(G–I)** Mitochondrial OCR analysis of different groups using the Seahorse XF24 Bioanalyzer device. **(J)** RT-PCR results of PGC-1*α* mRNA expression; **(K)** Western blotting results of PGC-1*α* protein expression. PGC-1*α* upregulation in ox-LDL treated HAECs reversed mitochondrial energy metabolism disorder. n.s., No significance.

PGC-1*α* shRNA expression plasmid was transfected into HAECs, and PGC-1*α* overexpression plasmid was transfected into ox-LDL treated HAECs to observe the changes of mitochondrial energy metabolism. The results showed that in HAECs transfected with PGC-1*α* shRNA, cell apoptosis and mitochondrial ROS levels were significantly increased, mitochondrial DNA copy number, complex I and III activity and ATP levels were significantly decreased, and basic and maximal OCRs were also decreased. In ox-LDL treated HAECs transfected with overexpressed PGC-1*α*, cell apoptosis and mitochondrial ROS levels significantly decreased, mitochondrial DNA copy number, complex I and III activity, ATP levels and basic and maximal OCRs significantly increased (**Figures 5A–I**). Transfection of PGC-1*α* shRNA into HAECs decreased the PGC-1*α* expression and *vice versa* (**Figures 5J, K**). These results suggested that upregulation of PGC-1*α* expression in ox-LDL treated HAECs reversed mitochondrial energy metabolism disorder.

### Dose Effect of PGC-1*α*

**Figure 6:**
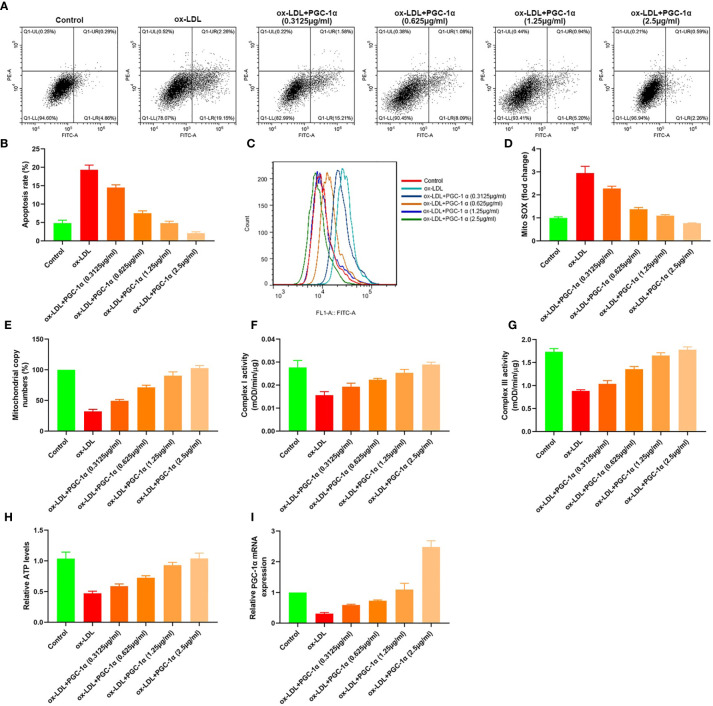
Dose effect of PGC-1*α.* **(A, B)** Apoptosis of HAECs detected by flow cytometry; **(C)** Mitochondrial ROS levels in HAECs detected by flow cytometry; **(D)** mito SOX levels; **(E)** RT-PCR results of the copy number of mtDNA in HAECs; **(F, G)** Mitochondrial complex I and III activity in HAECs; **(H)** Relative ATP content in HAECs; **(I)** Relative PGC-1*α* mRNA expression.

We transfected 7.5 μl (0.3125 μg/ml, 0.625 μg/ml, 1.25 μg/ml, and 2.5 μg/ml) PGC-1*α* eukaryotic expression vectors into HAECs treated with ox LDL, respectively. The apoptosis rates, mito SOX, mitochondrial copy numbers, Complex I activity, Complex III activity, and relative ATP levels were determined. The results showed that the apoptosis rates and mito Sox decreased with the increase of PGC-1*α* transfection, and the mitochondrial copy numbers, Complex I activity, Complex III activity and relative ATP levels increased with the increase of PGC-1*α* transfection (**Figures 6A–I**).

## Discussion

In this study, we found that ox-LDL inhibited the expression of LncRNA FENDRR in HAECs and downregulated the expression of PGC-1*α* through FENDRR/miR-18a-5p/PGC-1*α* signal axis, resulting in the increase of ROS level and the disorder of mitochondrial energy metabolism in HAECs.

VECs maintain intravascular homeostasis mainly by regulating vascular relaxation, leukocyte adhesion, platelet activation, and smooth muscle cell proliferation and migration (37, 38). The first pathophysiological change is endothelial dysfunction in the early stage of AS, which characterized by the decrease of NO synthesis and secretion (39). Endothelial nitric oxide (NO) inhibits the endothelial cell adhesion molecules and chemokine expression and inhibited platelet activation and aggregation. NO is synthesized by converting L-arginine to L-citrulline by endothelial nitric oxide synthase (eNOS) in endothelial cells (40). The main reason for the reduction of NO production is ROS mediated oxidative stress-induced eNOS degradation rather than the eNOS gene inhibition (41). eNOS dysfunction could produce more ROS, further damaged endothelial function, and promoted the AS development (40). The mitochondria are involved in ROS generation, apoptosis and intracellular signal transduction of VECs, and play a key role in the VEC function regulation (42). Excessive ROS production leads to endothelial cell senescence, apoptosis and AS. VEC dysfunction with mitochondrial damage and dysfunction preceded cardiovascular disease (43). Mitochondrial respiratory chain is the main pathway of energy production. Under pathological conditions, the effective utilization of respiratory chain decreased, ATP synthesis decreased, and more electrons were released to produce ROS (42). mtDNA is highly susceptible to ROS attack (44). mtDNA mitochondrial damage is an early event of AS in ApoE−/− mice (45). In this study, we used ox-LDL to treat HAECs and found that ATP synthesis decreased, mitochondrial OCRs, mtDNA copy number decreased, mitochondrial ROS level, and apoptosis level increased. Upregulation of FENDRR expression in ox-LDL treated HAECs could reverse the effect of ox-LDL and reduce the level of mitochondrial ROS and apoptosis. These results were consistent with the above studies, indicated that ox-LDL affected the mitochondrial function, and promoted apoptosis in HAECs by downregulating the expression of FENDRR.

The study confirmed that non-coding RNA was involved in a variety of cardiovascular diseases, including AS (46). In this study, ENCORI analysis showed that FENDRR/miR-18a-5p/PGC-1*α* signal axis may be involved in the damage of ox-LDL on mitochondrial energy metabolism of HAECs. RIP results showed that miR-18a-5p, FENDRR, and PGC-1*α* mRNA could bind directly. The results of luciferase reporter gene also indicated that there were targeted regulatory effects between FENDRR and miR-18a-5p, miR-18a-5p and PGC-1*α* 3′-UTR. In patients with hypertrophic cardiomyopathy, the circulating miR-18a-5p expression was significantly increased, which was positively correlated with the disease progression and could also be used as a biomarker of diffuse myocardial fibrosis (47). In this study, the miR-18a-5p expression was upregulated, and FENDRR expression was downregulated in ox-LDL treated HAECs. Transfection of FENDRR or miR-18a-5p inhibitor into ox-LDL treated HAECs could reverse the effect of ox-LDL and reduce the level of mitochondrial ROS and apoptosis. Transfection of miR-18a-5p mimics or sh-FENDRR in normal HAECs could cause mitochondrial energy metabolism disorder, upregulate mitochondrial ROS level and apoptosis level. These results suggested that FENDRR/miR-18a-5p/PGC-1*α* signal axis existed in HAECs, and ox-LDL caused mitochondrial energy metabolism disorder in HAECs by FENDRR/miR-18a-5p/PGC-1*α* signal axis.

PGC-1*α* is a main regulator of mitochondrial biosynthesis. PGC-1*α* activates mitochondrial transcription factors A and B, which regulate the mtDNA encoded genes’ expression, including the basic subunit of electron transport chain (48). PGC-1*α* significantly inhibits endothelium-dependent vasodilation induced by fatty acids and recovers the function of NO (49). PGC-1*α* repair fatty acid oxidation of endothelial cells, so as to improve the activity of ATP/ADP transporter, increase ATP synthesis, reduce ROS production, so that endothelial cells can adapt well with high lipid load (12). PGC-1*α* overexpression increased the UCP-2 and mitochondrial antioxidant enzymes’ expression, including catalase, manganese SOD, and thioredoxin 2, but silencing PGC-1*α* effect was the opposite (49). PGC-1*α* can also prevent the VECs’ apoptosis, limit the activation of inflammation, prevent the activation of c-Jun N-terminal kinase, and increase the bioavailability of NO (50). We found that the PGC-1*α* expression was decreased in ox-LDL treated HAECs. Overexpression of PGC-1*α* in ox-LDL treated HAECs could reverse the effect of ox-LDL and reduce the level of mitochondrial ROS and apoptosis. Downregulation of PGC-1*α* expression in normal HAECs could cause mitochondrial energy metabolism disorder, increase mitochondrial ROS level and apoptosis level. These results further confirmed that FENDRR/miR-18a-5p/PGC-1*α* signal axis is involved in the regulation of mitochondrial energy metabolism in HAECs.

## Conclusions

In conclusion, this study showed that FENDRR acted as a molecular sponge to adsorb miR-18a-5p in normal HAECs and inhibited the negative regulation of miR-18a-5p on PGC-1*α*, thus maintaining normal mitochondrial energy metabolism in HAECs. ox-LDL could downregulate the FENDRR expression in HAECs, decrease the expression of PGC-1*α*, cause the mitochondrial energy metabolism disorder, increase the level of ROS and apoptosis. This study presented the transcriptional regulation spectrum of key energy metabolism signal transduction pathways in vascular tissue and suggested potential therapeutic targets for vascular endothelial dysfunction related diseases. The data is too limited to ensure that the observed effects are due to direct regulatory events; further research is needed.

## Data Availability Statement

The raw data supporting the conclusions of this article will be made available by the authors, without undue reservation.

## Author Contributions

Conception and design: GW. Administrative support: GW. Provision of study materials or patients: GW and YY. Collection and assembly of data: HM and LS. Data analysis and interpretation: GW, WJ, XH, and WL. All authors contributed to the article and approved the submitted version.

## Conflict of Interest

The authors declare that the research was conducted in the absence of any commercial or financial relationships that could be construed as a potential conflict of interest.

## References

[1] Thompson RC Allam AH Lombardi GP Wann LS Sutherland ML Sutherland JD. Atherosclerosis across 4000 years of human history: the Horus study of four ancient populations. Lancet (2013) 381(9873):1211–22. 10.1016/S0140-6736(13)60598-X 23489753

[2] Frostegård J. Immunity, atherosclerosis and cardiovascular disease. BMC Med (2013) 11:117. 10.1186/1741-7015-11-117 23635324 PMC3658954

[3] Schaftenaar F Frodermann V Kuiper J Lutgens E. Atherosclerosis: the interplay between lipids and immune cells. Curr Opin Lipidol (2016) 27(3):209–15. 10.1097/MOL.0000000000000302 27031276

[4] Siti HN Kamisah Y Kamsiah J. The role of oxidative stress, antioxidants and vascular inflammation in cardiovascular disease (a review). Vascul Pharmacol (2015) 71:40–56. 10.1016/j.vph.2015.03.005 25869516

[5] Cai H Harrison DG. Endothelial dysfunction in cardiovascular diseases: the role of oxidant stress. Circ Res (2000) 87(10):840–4. 10.1161/01.RES.87.10.840 11073878

[6] Kattoor AJ Kanuri SH Mehta JL. Role of Ox-LDL and LOX-1 in Atherogenesis. Curr Med Chem (2019) 26(9):1693–700. 10.2174/0929867325666180508100950 29737246

[7] Chen JJ Tao J Zhang XL Xia LZ Zeng JF Zhang H. Inhibition of the ox-LDL-Induced Pyroptosis by FGF21 of Human Umbilical Vein Endothelial Cells Through the TET2-UQCRC1-ROS Pathway. DNA Cell Biol (2020) 39(4):661–70. 10.1089/dna.2019.5151 32101022

[8] Cadenas S. Mitochondrial uncoupling, ROS generation and cardioprotection. Biochim Biophys Acta Bioenerg (2018) 1859(9):940–50. 10.1016/j.bbabio.2018.05.019 29859845

[9] Suwa M Nakano H Kumagai S. Effects of chronic AICAR treatment on fiber composition, enzyme activity, UCP3, and PGC-1 in rat muscles. J Appl Physiol (1985) (2003) 95(3):960–8. 10.1152/japplphysiol.00349.2003 12777406

[10] Valle I Alvarez-Barrientos A Arza E Lamas S Monsalve M. PGC-1alpha regulates the mitochondrial antioxidant defense system in vascular endothelial cells. Cardiovasc Res (2005) 66(3):562–73. 10.1016/j.cardiores.2005.01.026 15914121

[11] Wang Q Li L Li CY Pei Z Zhou M Li N. SIRT3 protects cells from hypoxia via PGC-1α- and MnSOD-dependent pathways. Neuroscience (2015) 286:109–21. 10.1016/j.neuroscience.2014.11.045 25433241

[12] Won JC Park JY Kim YM Koh EH Seol S Jeon BH. Peroxisome proliferator-activated receptor-gamma coactivator 1-alpha overexpression prevents endothelial apoptosis by increasing ATP/ADP translocase activity. Arterioscler Thromb Vasc Biol (2010) 30(2):290–7. 10.1161/ATVBAHA.109.198721 19965780

[13] Chen LL. Linking Long Noncoding RNA Localization and Function. Trends Biochem Sci (2016) 41(9):761–72. 10.1016/j.tibs.2016.07.003 27499234

[14] Stuwe E Toth KF Aravin AA. Small but sturdy: small RNAs in cellular memory and epigenetics. Genes Dev (2014) 28(5):423–31. 10.1101/gad.236414.113 PMC3950340 24589774

[15] Duval M Cossart P Lebreton A. Mammalian microRNAs and long noncoding RNAs in the host-bacterial pathogen crosstalk. Semin Cell Dev Biol (2017) 65:11–9. 10.1016/j.semcdb.2016.06.016 PMC7089780 27381344

[16] Paraskevopoulou MD Hatzigeorgiou AG. Analyzing MiRNA-LncRNA Interactions. Methods Mol Biol (2016) 1402:271–86. 10.1007/978-1-4939-3378-5_21 26721498

[17] Ferrè F Colantoni A Helmer-Citterich M. Revealing protein-lncRNA interaction. Brief Bioinform (2016) 17(1):106–16. 10.1093/bib/bbv031 PMC4719072 26041786

[18] Kumar S Williams D Sur S Wang JY Jo H. Role of flow-sensitive microRNAs and long noncoding RNAs in vascular dysfunction and atherosclerosis. Vascul Pharmacol (2019) 114:76–92. 10.1016/j.vph.2018.10.001 30300747 PMC6905428

[19] Hulshoff MS Del Monte-Nieto G Kovacic J Krenning G. Non-coding RNA in endothelial-to-mesenchymal transition. Cardiovasc Res (2019) 115(12):1716–31. 10.1093/cvr/cvz211 PMC6755356 31504268

[20] Zhang HN Xu QQ Thakur A Alfred MO Chakraborty M Ghosh A. Endothelial dysfunction in diabetes and hypertension: Role of microRNAs and long non-coding RNAs. Life Sci (2018) 213:258–68. 10.1016/j.lfs.2018.10.028 30342074

[21] Papait R Kunderfranco P Stirparo GG Latronico MV Condorelli G. Long noncoding RNA: a new player of heart failure? J Cardiovasc Transl Res (2013) 6(6):876–83. 10.1007/s12265-013-9488-6 PMC3838575 23835777

[22] Grote P Wittler L Hendrix D Koch F Währisch S Beisaw A. The tissue-specific lncRNA Fendrr is an essential regulator of heart and body wall development in the mouse. Dev Cell (2013) 24(2):206–14. 10.1016/j.devcel.2012.12.012 PMC4149175 23369715

[23] Çekin N Özcan A Göksel S Arslan S Pınarbaşı E Berkan Ö. Decreased FENDRR and LincRNA-p21 expression in atherosclerotic plaque. Anatol J Cardiol (2018) 19(2):131–6. 10.14744/AnatolJCardiol.2017.8081 PMC5864808 29424733

[24] Xu TP Huang MD Xia R Liu XX Sun M Yin L. Decreased expression of the long non-coding RNA FENDRR is associated with poor prognosis in gastric cancer and FENDRR regulates gastric cancer cell metastasis by affecting fibronectin1 expression. J Hematol Oncol (2014) 7:63. 10.1186/s13045-014-0063-7 25167886 PMC4237812

[25] Zhang G Wang Q Zhang X Ding Z Liu R. LncRNA FENDRR suppresses the progression of NSCLC via regulating miR-761/TIMP2 axis. BioMed Pharmacother (2019) 118:109309. 10.1016/j.biopha.2019.109309 31545237

[26] Liu J Du W. LncRNA FENDRR attenuates colon cancer progression by repression of SOX4 protein. Onco Targets Ther (2019) 12:4287–95. 10.2147/OTT.S195853 PMC6549791 31213846

[27] Kun-Peng Z Xiao-Long M Chun-Lin Z. LncRNA FENDRR sensitizes doxorubicin-resistance of osteosarcoma cells through down-regulating ABCB1 and ABCC1. Oncotarget (2017) 8(42):71881–93. 10.18632/oncotarget.17985 PMC5641097 29069754

[28] Zhang G Han G Zhang X Yu Q Li Z Li Z. Long non-coding RNA FENDRR reduces prostate cancer malignancy by competitively binding miR-18a-5p with RUNX1. Biomarkers (2018) 23(5):435–45. 10.1080/1354750X.2018.1443509 29465000

[29] Shi Y Chen C Xu Y Liu Y Zhang H Liu Y. LncRNA FENDRR promotes high-glucose-induced proliferation and angiogenesis of human retinal endothelial cells. Biosci Biotechnol Biochem (2019) 83(5):869–75. 10.1080/09168451.2019.1569499 30700211

[30] Qin X Lu M Zhou Y Li G Liu Z. LncRNA FENDRR represses proliferation, migration and invasion through suppression of survivin in cholangiocarcinoma cells. Cell Cycle (2019) 18(8):889–97. 10.1080/15384101.2019.1598726 PMC6527288 30983519

[31] Wang B Xian J Zang J Xiao L Li Y Sha M. Long non-coding RNA FENDRR inhibits proliferation and invasion of hepatocellular carcinoma by down-regulating glypican-3 expression. Biochem Biophys Res Commun (2019) 509(1):143–7. 10.1016/j.bbrc.2018.12.091 30573358

[32] Zhang F Ni H Li X Liu H Xi T Zheng L. LncRNA FENDRR attenuates adriamycin resistance via suppressing MDR1 expression through sponging HuR and miR-184 in chronic myelogenous leukaemia cells. FEBS Lett (2019) 593(15):1993–2007. 10.1002/1873-3468.13480 31180580

[33] Li JH Liu S Zhou H Qu LH Yang JH. starBase v2.0: decoding miRNA-ceRNA, miRNA-ncRNA and protein-RNA interaction networks from large-scale CLIP-Seq data. Nucleic Acids Res (2014) 42(Database issue):D92–7. 10.1093/nar/gkt1248 PMC3964941 24297251

[34] Yadav H Quijano C Kamaraju AK Gavrilova O Malek R Chen W. Protection from obesity and diabetes by blockade of TGF-β/Smad3 signaling. Cell Metab (2011) 14(1):67–79. 10.1016/j.cmet.2011.04.013 21723505 PMC3169298

[35] Manfredi G Yang L Gajewski CD Mattiazzi M. Measurements of ATP in mammalian cells. Methods (2002) 26(4):317–26. 10.1016/S1046-2023(02)00037-3 12054922

[36] Ayanga BA Badal SS Wang Y Galvan DL Chang BH Schumacker PT. Dynamin-Related Protein 1 Deficiency Improves Mitochondrial Fitness and Protects against Progression of Diabetic Nephropathy. J Am Soc Nephrol (2016) 27(9):2733–47. 10.1681/ASN.2015101096 PMC5004662 26825530

[37] Szewczyk A Jarmuszkiewicz W Koziel A Sobieraj I Nobik W Lukasiak A. Mitochondrial mechanisms of endothelial dysfunction. Pharmacol Rep (2015) 67(4):704–10. 10.1016/j.pharep.2015.04.009 26321271

[38] Wang Q Zhao T Zhang W Yu W Liu B Wang Z. Poly (ADP-Ribose) Polymerase 1 Mediated Arginase II Activation Is Responsible for Oxidized LDL-Induced Endothelial Dysfunction. Front Pharmacol (2018) 9:882. 10.3389/fphar.2018.00882 30158868 PMC6104189

[39] Victor VM Apostolova N Herance R Hernandez-Mijares A Rocha M. Oxidative stress and mitochondrial dysfunction in atherosclerosis: mitochondria-targeted antioxidants as potential therapy. Curr Med Chem (2009) 16(35):4654–67. 10.2174/092986709789878265 19903143

[40] Pircher A Treps L Bodrug N Carmeliet P. Endothelial cell metabolism: A novel player in atherosclerosis? Basic principles and therapeutic opportunities. Atherosclerosis (2016) 253:247–57. 10.1016/j.atherosclerosis.2016.08.011 27594537

[41] Frey RS Gao X Javaid K Siddiqui SS Rahman A Malik AB. Phosphatidylinositol 3-kinase gamma signaling through protein kinase Czeta induces NADPH oxidase-mediated oxidant generation and NF-kappaB activation in endothelial cells. J Biol Chem (2006) 281(23):16128–38. 10.1074/jbc.M508810200 16527821

[42] Chistiakov DA Shkurat TP Melnichenko AA Grechko AV Orekhov AN. The role of mitochondrial dysfunction in cardiovascular disease: a brief review. Ann Med (2018) 50(2):121–7. 10.1080/07853890.2017.1417631 29237304

[43] Guo C Wang J Jing L Ma R Liu X Gao L. Mitochondrial dysfunction, perturbations of mitochondrial dynamics and biogenesis involved in endothelial injury induced by silica nanoparticles. Environ Pollut (2018) 236:926–36. 10.1016/j.envpol.2017.10.060 29074197

[44] Yu EP Bennett MR. The role of mitochondrial DNA damage in the development of atherosclerosis. Free Radic Biol Med (2016) 100:223–30. 10.1016/j.freeradbiomed.2016.06.011 27320189

[45] Mercer JR Cheng KK Figg N Gorenne I Mahmoudi M Griffin J. DNA damage links mitochondrial dysfunction to atherosclerosis and the metabolic syndrome. Circ Res (2010) 107(8):1021–31. 10.1161/CIRCRESAHA.110.218966 PMC2982998 20705925

[46] Stojkovic S Nossent AY Haller P Jäger B Vargas KG Wojta J. MicroRNAs as Regulators and Biomarkers of Platelet Function and Activity in Coronary Artery Disease. Thromb Haemost (2019) 119(10):1563–72. 10.1055/s-0039-1693702 31421643

[47] Fang L Ellims AH Moore XL White DA Taylor AJ Chin-Dusting J. Circulating microRNAs as biomarkers for diffuse myocardial fibrosis in patients with hypertrophic cardiomyopathy. J Transl Med (2015) 13:314. 10.1186/s12967-015-0672-0 26404540 PMC4581079

[48] Kluge MA Fetterman JL Vita JA. Mitochondria and endothelial function. Circ Res (2013) 112(8):1171–88. 10.1161/CIRCRESAHA.111.300233 PMC3700369 23580773

[49] Geng T Li P Yin X Yan Z. PGC-1α promotes nitric oxide antioxidant defenses and inhibits FOXO signaling against cardiac cachexia in mice. Am J Pathol (2011) 178(4):1738–48. 10.1016/j.ajpath.2011.01.005 PMC3078433 21435455

[50] Martínez-Redondo V Pettersson AT Ruas JL. The hitchhiker’s guide to PGC-1α isoform structure and biological functions. Diabetologia (2015) 58(9):1969–77. 10.1007/s00125-015-3671-z 26109214

