# Supplementary material for: LncRNA FENDRR Inhibits ox-LDL Induced Mitochondrial Energy Metabolism Disorder in Aortic Endothelial Cells via miR-18a-5p/PGC-1α Signaling Pathway
Source: Front Endocrinol (Lausanne). 2021 Apr 12;12:622665. doi: 10.3389/fendo.2021.622665 (PMC8072360; doi:10.3389/fendo.2021.622665)
Supplement: Supplementary file 1 [file DataSheet_1.docx]

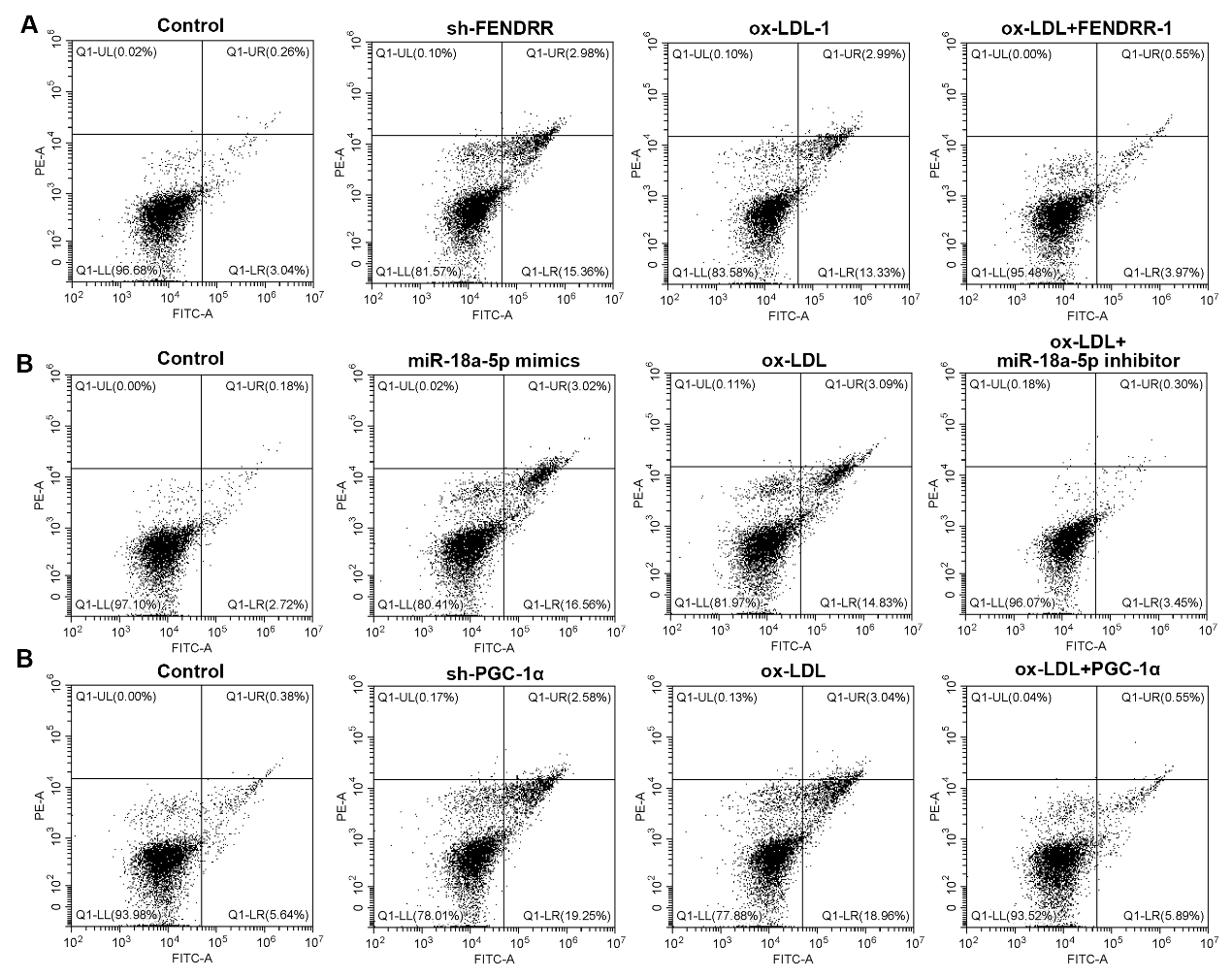


Supplemental Figure The data of cell apoptosis by flow cytometry in this study

**sh-FENDRR：**

Sense: 5’- GCGAUUGACUGUCUUAUAATT -3’

Antisense: 5’-UUAUAAGACAGUCAAUCGCTT -3’

**sh-PGC-1α：**

Sense: 5’- GCGAAUCCAGUUUGUGCAATT -3’

Antisense: 5’-UUGCACAAACUGGAUUCGCTT -3’

**sh-NC：**

Sense: 5’- UUCUCCGAACGUGUCACGUTT -3’

Antisense: 5’-ACGUGACACGUUCGGAGAATT-3’

**hsa-miR-18a-5p Inhibitor**

5’-CUAUCUGCACUAGAUGCACCUUA-3’

**hsa-miR-18a-5p mimics**

5’-UAAGGUGCAUCUAGUGCAGAUAG-3’

**NC：**

5’-CAGUACUUUUGUGUAGUACAA-3’
