# Supplementary figures and images for: LncRNA FENDRR Inhibits ox-LDL Induced Mitochondrial Energy Metabolism Disorder in Aortic Endothelial Cells via miR-18a-5p/PGC-1α Signaling Pathway
Source: Front Endocrinol (Lausanne). 2021 Apr 12;12:622665. doi: 10.3389/fendo.2021.622665 (PMC8072360; doi:10.3389/fendo.2021.622665)

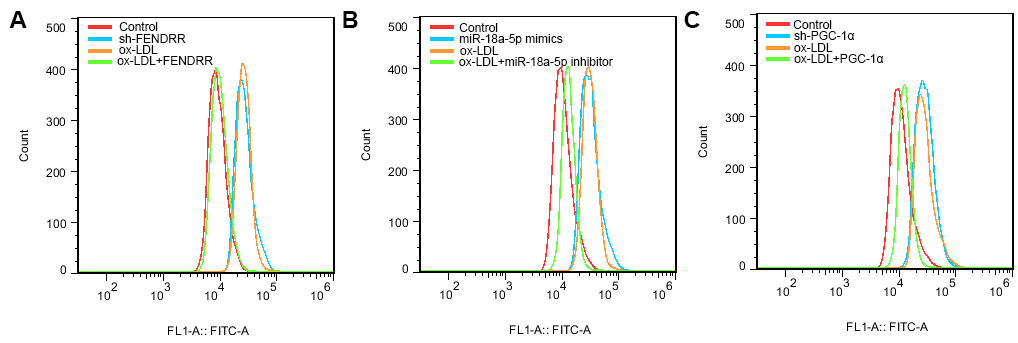

Supplement: Supplementary file 2 [file Image_1.jpg]
